# Hæmophilia

**Published:** 1905-07-08

**Authors:** 


### HÆMOPHILIA.

In a recent paper on this mysterious disease, Dr. F. P. Kinnicutt[1] takes for his text the case of a patient of his whose family history was known for several generations back, and who was thus a favourable subject for investigation as regards inherited proclivities. The patient was a boy of 16, four generations of whose ancestors on both sides had never shown any signs of hæmophilia, while his mother had borne four children—three boys and one girl—none of whom were bleeders except the patient. The personal history of the latter showed that he was liable to easy bruising from the age of five months; that at two years of age he suffered from a severe epistaxis, and at five began the first of a series of so-called rheumatic attacks, which were no doubt the recognised arthritic phenomena of hæmophilia. Thenceforward he was the victim of numerous attacks of joint hæmorrhage associated with a moderate degree of fever, the attacks increasing in severity with their number, while bleeding from mucous surfaces, such as those of the nose and intestine, was a common occurrence. In his 21st year large interstitial hæmorrhages took place in the muscles of the extremities, and later in the year the abdominal wall was similarly affected. During the next two years the repeated arthritic lesions left his knee-joints the seat of a chronic thickening; the movements of the joints gradually became gravely impaired, and ultimately the appearance of contractures compelled the use of crutches. One day while getting about on his crutches he was seized with an acute pain in the abdomen, which passed off gradually, leaving a palpable mass, undoubtedly a hæmatoma, in the hypogastric regions. Six weeks later a recurrence of the pain was accompanied by signs of grave concealed bleeding, and he died of syncope probably due to intestinal hæmorrhage, though no autopsy was permitted by his relatives. He died in his 23rd year.

Although in this case the family history was unorthodox, the laws governing the transmission of the hæmophilic tendency are fairly well established. They are stated by the author as follows:

1. As a rule the daughters of a bleeder-father are exempt from the disease, but transmit it to their male offspring.
2. The sons are also as a rule exempt, and do not transmit it to their offspring.
3. The daughter of a bleeder-father may transmit the disease to one, or several, or all her offspring.
4. Where there are several daughters the capacity for transmission may be confined to any fraction of their number.
5. Occasionally there is a direct transmission from father to son through several generations.
6. The disease does not appear in the issue of the sons of bleeder-families who are not themselves bleeders.

Dealing more particularly with the joint-lesions of the disease the author has collected cases showing that they may appear even in early infancy; it appears, moreover, that after adolescence, bleeders who survive the accidents of the disease become progressively less prone to arthritic hæmorrhages, however severe may have been their past experience in this particular. The clinical aspect of an arthritic hæmorrhage is that of a rapid effusion, generally into the knee-joints, accompanied by varying degrees of pain, and unmarked by any discolouration of the overlying skin.

Dr. Kinnicutt summarises as follows the therapeutics most in favour:—

1. Chloride of calcium in doses of 30 grains twice or thrice daily. The experiments of Professor Wright show that while the administration of this drug increases the coagulability of the blood of hæmophilic patients within a few hours of its ingestion, a continued use of it for several days is unable to maintain the increased coagulability. An important point in its exhibition, therefore, is that it should be intermittently given, an interval of about 24 hours being observed every two or three days.
2. The same salt, in solutions of a strength of one-half per cent. for accessible surface bleedings.
3. The addition to the foregoing solution of one-sixth of its volume of an aqueous extract containing nucleo-albumin, for use as a local application. The aqueous extract may be prepared from testicle, thyroid, thymus, or ovary, pounded in a weak alkaline liquid, and filtered.
4. The application of a stream of carbonic acid gas in an accessible hæmorrhage, and, in the case of a concealed one, its inhalation, in free admixture with ordinary air or oxygen.
5. The use of a 4 per cent. solution of cocaine hydrate, or of adrenalin, 1 to 1,000, with compression in cases of surface bleedings. Dr. Kinnicutt's contribution, though it adds nothing dramatic to our knowledge of hæmophilia, provides an excellent account of this intractable disease, and has an enhanced value for scientific purposes from the voluminous bibliography appended to it.

[1] Med. Record (N.Y.), June 10, 1905.

